# Long-term outcome of spiral ileal neobladder with orthotopic ureteral reimplantation

**DOI:** 10.1007/s11255-019-02296-x

**Published:** 2019-09-27

**Authors:** Huan Zhong, Yuefan Shen, Zixiang Yao, Xiaonong Chen, Jianguo Gao, Anping Xiang, Weigao Wang

**Affiliations:** Department of Urology, The First People’s Hospital of Huzhou, The First Affiliated Hospital of Huzhou Teachers College, No. 158, Guangchanghou Road, Huzhou, 313000 Zhejiang China

**Keywords:** Ileal neobladder, Orthotopic ureteral reimplantation, Urinary diversion, Radical cystectomy

## Abstract

**Purpose:**

The purpose of this study was to analyze long-term complications, urodynamics, and quality of life (QoL) of patients after orthotopic ileal neobladder with orthotopic ureteral reimplantation to enrich clinical data and provide a basis for clinical use of this surgery.

**Methods:**

Between January 2007 and January 2013, 72 consecutive patients who underwent spiral ileal neobladder following radical cystectomy were enrolled. The neobladder was created using a modified Camey-II technique. Complications were reviewed and staged according to Clavien–Dindo classification and evaluated in long-term follow-up. Urodynamics were performed, and QoL was assessed by the Functional Assessment of Cancer Therapy for Bladder Cancer (FACT-BL) instrument.

**Results:**

The total follow-up time was 60 months, and the total survival rates at 3 and 5 years after surgery were 76.4% (55/72) and 65.3% (47/72), respectively. There were 34 (47.2%) early complications in 23 (31.9%) patients and 42 (58.3%) late complications in 35 (48.6%) patients. The total satisfactory control rates were 69.1% and 66.0% at 3 and 5 years after the surgery, respectively. Urodynamic studies were performed in some patients, and the receiver operating characteristic curve analysis showed that pressure at maximum capacity, compliance, and post void residual urine had predictive value for mortality (*P* < 0.05). The total FACT-BL scores of patients at 1, 3, and 5 years postoperation were 125.0 ± 15.2, 127.0 ± 16.2, and 120.6 ± 13.5, respectively, and it decreased at 5 years postoperation (*P* < 0.05).

**Conclusion:**

Spiral ileal neobladder with orthotopic ureteral reimplantation offers satisfactory long-term results, and urodynamic monitoring might have prognostic value.

## Introduction

Global Cancer Statistics 2018 predicts that there will be 549,393 new cancer cases and 199,922 deaths due to bladder cancer worldwide in 2018 [1]. The high prevalence of bladder cancer, together with its vulnerability to multiple recurrences and progression despite local therapy, leads to a substantial economic burden on health services [2]. At present, radical cystectomy with urinary diversion is the best treatment option for patients with invasive bladder cancer. The development of urinary diversion mainly includes three stages: incontinent urinary diversion, continent urinary diversion, and neobladder. In recent decades, ileal neobladder has become the most common urinary diversion method because it can provide a better quality of life (QoL). With the advantage of autonomic urination and no requirement for abdominal wall ostomy, ileal neobladder motivates more patients to accept radical cystectomy earlier in the disease process [3]. However, the ileal neobladder surgery is complicated and difficult to master, leading to a few inevitable complications, which affect the prognosis of patients.

Although previous studies have revealed that patients undergoing ileal neobladder surgery had better QoL than those who underwent ileal conduit [4], most of the patients who chose neobladder were younger and had less severe illness [5]. Because very few randomized controlled trials have compared the advantages of ileal neobladder surgery and traditional ileal conduit, it remains controversial whether the long-term QoL and renal function of patients after ileal neobladder surgery are better than those after traditional ileal conduit [6]. Moreover, the indications for ileal neobladder surgery are strictly limited, and the cardiopulmonary function, blood glucose level, intestinal health status, and compliance of patients need to be strictly screened before treatment. To adequately counsel patients, accurate data regarding the adverse events, postoperative function, and long-term life quality of different types of urinary diversion are required. In the present study, we retrospectively analyzed the clinical and follow-up data of 72 patients after they underwent spiral orthotopic ileal neobladder to enrich the clinical data and provide a basis for the clinical use of ileal neobladder.

## Materials and methods

### Study population

This study was approved by the ethics committee of The First People’s Hospital of Huzhou, and all patients provided written informed consent and underwent clinical and laboratory evaluations before surgery. Seventy-two consecutive male patients with bladder cancer who underwent radical cystectomy in The First People’s Hospital of Huzhou between January 2007 and January 2013 were selected for this study. All patients underwent pelvic MRI or enhanced CT examination before surgery, and they were confirmed to have muscle-invasive bladder cancer by both cystoscopy biopsy before surgery and postoperative pathology. No urothelial carcinoma was found in the neck of the bladder. Patients with the following conditions were excluded from the study: serum creatinine > 200 pmol/L, severe impaired liver function, urinary stress incontinence, inflammatory bowel disease, tumor infiltration of the distal prostatic urethra in men, inadequate intellectual capacity, and neurological and psychotic disorders. The clinical and pathologic characteristics of patients are shown in Table 1.

**Table 1:** Clinical and pathologic characteristics of patients receiving orthotopic ileal neobladder

Characteristics	Patients
Age [median (IQR)]	62 (41, 72)
BMI (kg/m^2^)	23.22 ± 2.06
ECOG score [median (IQR)]	1 (1, 2)
Pathologic category [*n* (%)]	
Uroepithelium carcinoma	65 (90.28)
Adenocarcinoma	4 (5.56)
Squamous carcinoma	3 (4.17)
Basic disease [*n* (%)]	
Cardiovascular disease	7 (9.72)
Diabetes mellitus	3 (4.17)
Hypertension	5 (6.94)
Smoking history [*n* (%)]	13 (18.06)
Intact erectile function [*n* (%)]	30 (41.67)
Neoadjuvant chemotherapy history [*n* (%)]	10 (13.89)
TMN stage [*n* (%)]	
T1N0M0	7 (9.72)
T2N0M0	17 (23.61)
T3N0M0	26 (36.11)
T3N1M0	19 (26.39)
T4N1M0	3 (4.17)
T3N2M1	3 (4.17)
WHO grade	
Low	6 (8.33)
High	66 (91.67)

*BMI* body mass index, *ECOG* Eastern Cooperative Oncology Group performance status, *TMN* tumor-node-metastasis, *IQR* interquartile range, *WHO* World Health Organization

### Surgical methods

**Fig. 1:**
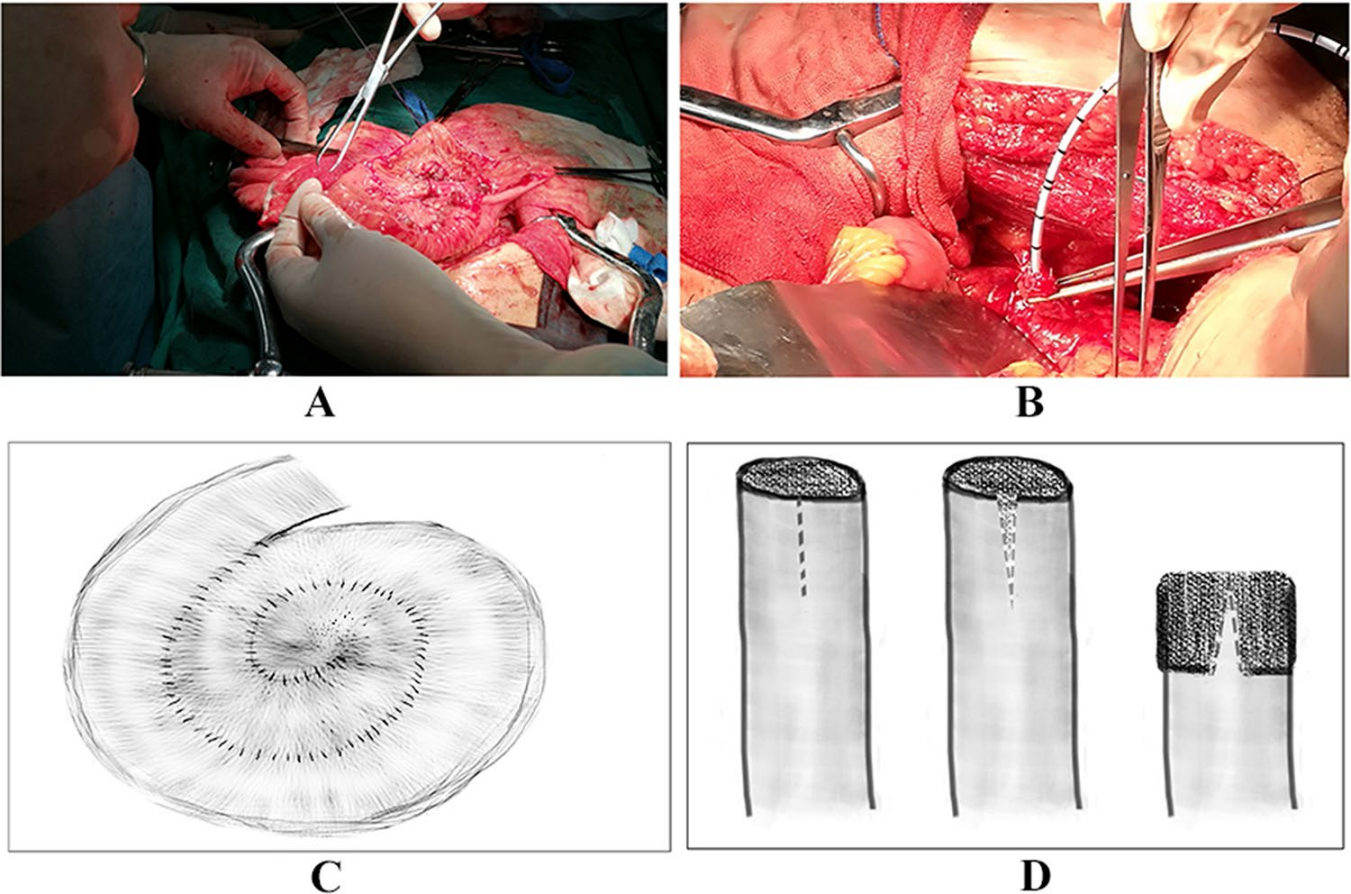
The “spiral-shaped” neobladder and “sleeve-shaped” ureter. **a** During the surgery, the prepared ileal segment was closed in a “spiral-shaped” manner to construct the neobladder; **b** during the surgery, the end of the ureter was split 1 cm and everted in a “sleeve shape” for anti-refluxing; **c** a sketch map of the “spiral-shaped” neobladder; **d** a sketch map of the “sleeve-shaped” ureter

All operations were performed by a single surgeon. Radical cystectomy with pelvic lymphadenectomy was performed according to the guidelines established by (The National Institute for Health and Care Excellence) NICE [7]. A 40- to 45-cm-long ileal segment was isolated 30 cm proximal to the ileocecal valve, and the bowel continuity was then restored by a single-layer running suture. The ileal segment was dissected along its entire length of the contralateral border of the mesentery, and the mucus from the surface of the intestinal mucosa was cleared by an aspirator. In this step, surface mucus cells of the intestinal tract are sufficiently destroyed by anhydrous ethanol. To construct the reservoir, the prepared ileal segment was closed in a “spiral-shaped” manner (Fig. 1a, c). The end of the ureter was split 1 cm and everted in a “sleeve shape” for antirefluxing (Fig. 1b, d), and it was then anastomosed to the reservoir using a ureteral stent. To complete this procedure, two intestinal incisions slightly larger than the ureteral circumference were made at the contralateral edge of the bilateral mesangium intestine at the top of the new bladder, and the ureteral papilla and ureteral stent were clamped and pulled into the new bladder with noninvasive forceps, ensuring that the ureteral nipple and stent were located in the new bladder. This process was the same as that of the ureter entering the bladder. The ureter was anastomosed to the new bladder with a 4-0 absorbable line using the split-cuff nipple nonrefluxing technique. Ureteral stents were exposed through the reservoir and the anterior abdominal wall. The lowest end of the new bladder was cut 1 cm to insert an 18F urethral catheter, and a 2-0 absorbable line was used to suture the corresponding sites (2, 5, 7, and 10 o’clock positions) at the bottom of the new bladder for four stitches. After a suprapubic catheter was placed into the new bladder through the fat of the mesoileum, the pouch was closed completely. Ureteral stents were removed 7–10 days later, and the suprapubic catheter was removed 2 weeks later. The urethral catheter was removed 16–21 days after the operation to restore urethral urination.

### Follow-up

A 5-year follow-up was completed after the surgery, and the patients were reexamined every 3 months for the first year, every 6 months for the second year, and every 12 months for the third to fifth year after the surgery. The main parameters assessed or recorded were as follows: (1) early complications (≤ 3 months) and late complications (> 3 months) were observed and classified by the Clavien–Dindo classification score (CCS) [8]. (2) Intravenous urographies were performed 3 months after the surgery. (3) A computed tomography (CT) scan of the thorax and abdomen was performed once a year to observe the morphology of the new bladder and the upper urinary tract. (4) Urinary control function was assessed by a bladder diary recorded by the patient himself. Three grades of urinary function were recorded: complete autonomic control, satisfactory control, and unsatisfactory control. Complete autonomic control was defined as complete urination control during the day and at night without the need for a urinal pad. Satisfactory control was defined as the need for only one pad during the day or at night. Unsatisfactory control was defined as requiring two pads or more during the day or at night. (5) Urodynamic studies were performed once a year. (6) HR-QoL was assessed by the Functional Assessment of Cancer Therapy for Bladder Cancer (FACT-BL) instrument in every re-examination.

### Statistical analysis

Statistical analyses were performed using SPSS software (version 22.0). All normally distributed quantitative variables are expressed as mean ± SD, while the remaining variables are expressed as median (interquartile range [IQR]) values. Receiver operating characteristic (ROC) curve analysis was used to ascertain the predicted value of urodynamics for death within 5 years after the surgery. Kaplan–Meier and log-rank tests were used to compare the survival time between patients with different levels of urodynamics. Differences with a two-tailed *P* value of less than 0.05 were considered statistically significant.

## Results

### Basic condition during operation

**Fig. 2:**
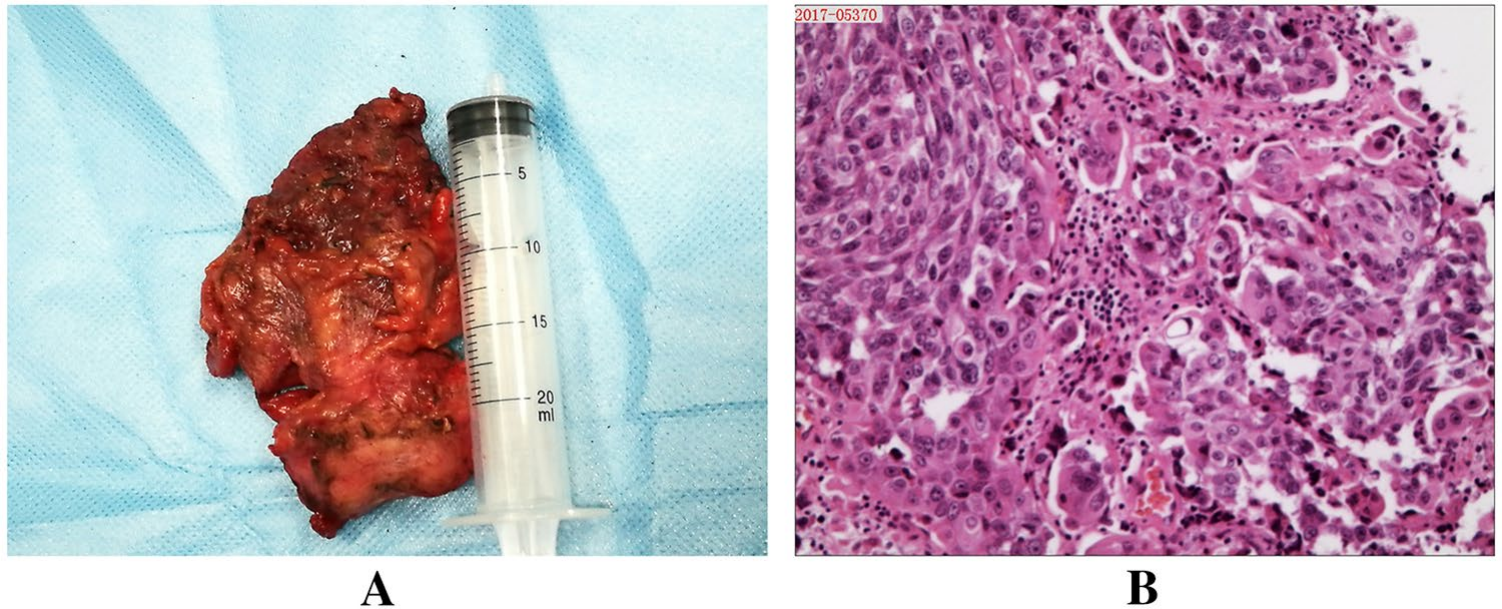
Pathology results of a typical case in the follow-up. **a** Postoperative resection of a cancerous tissue; **b** hematoxylin–eosin staining showed typical features of urothelial carcinoma

**Fig. 3:**
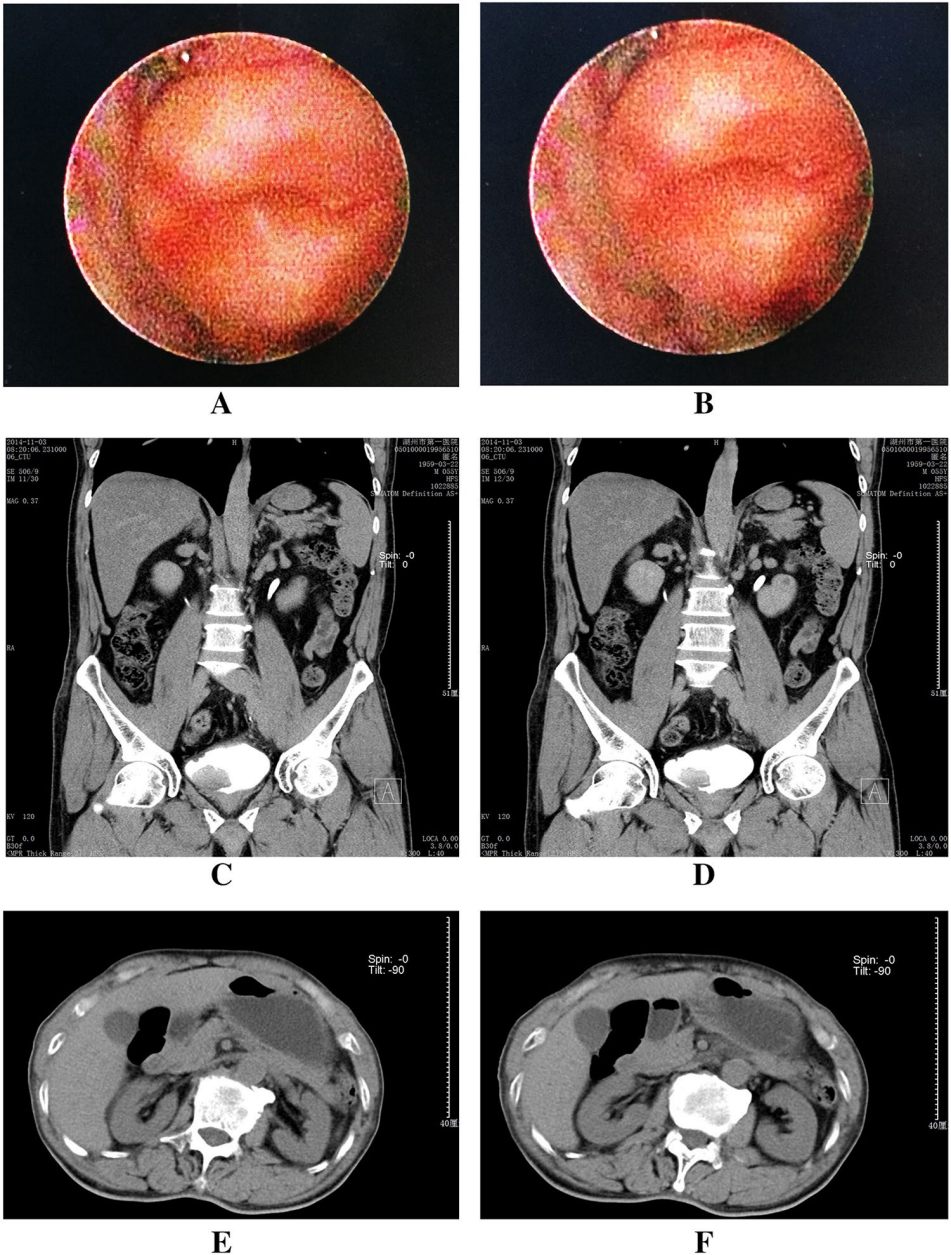
Imaging examination results of a typical case in the follow-up. **a**, **b** Cystoscopy examination results showed good ureteral opening; **c**, **d** frontal planes of intravenous pyelography examination results showed no hydronephrosis; **e**, **f** transverse planes of intravenous pyelography examination results showed no hydronephrosis

All patients completed the surgery without massive hemorrhage, and no perioperative deaths occurred. The operative time was 305.7 ± 37.2 min (range 235–342), the estimated blood loss was 802.3 ± 214.7 mL (range 505–1104), the gastrointestinal function recovery time was 3.8 ± 1.6 days (range 3–5), and the hospital stay time of patients was 22.9 ± 3.3 days (range 18–27). Figures 2 and 3 show imaging examination results of a typical case in the follow-up.

### Complications

A follow-up for 5 years after the surgery was performed. The total survival rate at 3 years and 5 years after surgery was 76.4% (55/72) and 65.3% (47/72). In 3 months after the surgery, there were 34 (47.2%) early complications in 23 (31.9%) patients, including 8 major complications (CCS III–V, 11.1%) and 26 minor complications (CCS I–II, 36.1%). In 60 months after the surgery, there were 42 (58.3%) late complications in 35 (48.6%) patients, including 33 major complications (CCS III–V, 45.8%) and nine minor complications (CCS I–II, 12.5%). Tables 2 and 3 provide an overview of the management of these complications.

**Table 2:** Early complications of patients following spiral orthotopic ileal neobladder

Early complications	*N* (%)	CCS grade	Treatment
Pouch-related			
Urosepsis	1 (1.39)	IVb	ICU
Ureteroneovesical stenosis	2 (2.78)	IIIb	Transurethral surgery
Enteroneovesical fistula	1 (1.39)	IIIb	Open surgery
Neovesicocutaneus fistula	1 (1.39)	IIIa	Endoscopic examination and antibiotics
Urinary tract infection	9 (12.50)	II	Antibiotics
Clot retention	2 (2.78)	II	Antibiotics
Urine leakage	2 (2.78)	II	Antibiotics and drainage
Urinoma	2 (2.78)	I	Prolonged drainage
Non-pouch-related			
Small intestine obstruction	3 (4.17)	IIIb	Intestinal anastomosis
Catheter‐related infections	3 (4.17)	II	Antibiotics
Pneumonia	2 (2.78)	II	Antibiotics
Deep venous thrombosis	1 (1.39)	II	Anticoagulants
Diarrhea	5 (6.94)	I	Nutrition and keeping balanced water and electrolytes

**Table 3:** Late complications of patients following spiral orthotopic ileal neobladder

Late complications	*N* (%)		Treatment
Pouch-related			
Ileourethral stenosis	13 (18.06)	IIIb	Transurethral surgery or open surgery
Neobladder calculus	3 (4.17)	IIIb	Transurethral lithotripsy
Recurrent urinary tract infection	11 (15.28)	II	Antibiotics
Nocturnal incontinence	4 (5.56)	II	Conservative therapy
Non-pouch-related			
Small intestine obstruction	4 (5.56)	IIIb	Intestinal anastomosis
Inguinal hernia	3 (4.17)	IIIb	ESWL
Secondary renal calculus	2 (2.78)	II	Conservative therapy
Hemorrhoid	2 (2.78)	II	Conservative therapy
Secondary renal calculus	2 (2.78)	II	Conservative therapy
Hemorrhoid	2 (2.78)	II	Conservative therapy

### Urinary status and urodynamics

**Fig. 4:**
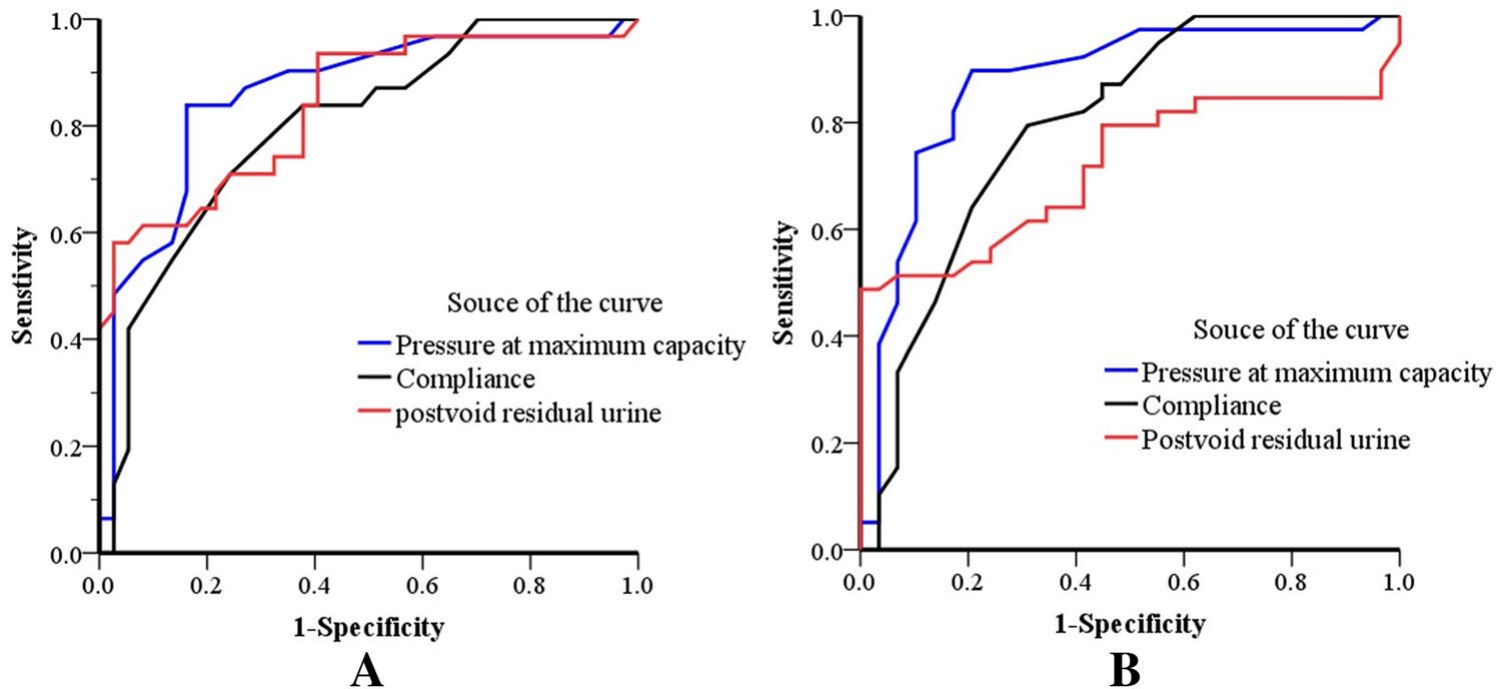
ROC curve of urodynamics to predict death at 3 and 5 years after surgery. **a** ROC curve of urodynamics to predict death at 3 years after surgery. The area under the curve (AUC) was 0.859, 0.800, and 0.837 for pressure at maximum capacity, compliance, and post void residual urine, respectively, *P *< 0.001; **b** ROC curve of urodynamics to predict death at 5 years after surgery. The AUC was 0.875, 0.796, and 0.717 for pressure at maximum capacity, urinary compliance, and post void residual urine, respectively, *P *< 0.001

As shown in Table 4, urinary incontinence was common in 3 months after surgery, and the total satisfactory control rate was only 48.6% (35/72); however, the continence rate increased substantially over time. By 1 year of follow-up, the total satisfactory control rate was 72.9% (52/70), and none of the patients required pads at daytime. Nighttime urinary incontinence was more prevalent in long-term follow-up, and the total satisfactory control rate was 69.1% and 66.0% at 3 years and 5 years after the surgery, respectively. In addition, urodynamic studies were performed in half of the patients (Table 5). To better understand the relationship between urodynamics and survival, we analyzed the predictive ability of urodynamics at 1 year after the surgery for mortality. The ROC curve (Fig. 4) showed that pressure at maximum capacity ([area under the curve (AUC)] = 0.859 and 0.875), compliance (AUC = 0.800 and 0.796), and post void residual urine (AUC = 0.837 and 0.717) had predictive value for mortality at 3 and 5 years after surgery (*P* < 0.05), respectively.

**Table 4:** Urinary status of patients following spiral orthotopic ileal neobladder

Time	*N*	Complete autonomic control	Satisfactory control	Unsatisfactory control	Total satisfactory control rate
3 months after the surgery	72	9 (12.5)	26 (36.1)	37 (51.4)	35 (48.6)
1 year after the surgery	70	22 (31.4)	30 (42.9)	18 (25.7)	52 (74.3)
3 years after the surgery	55	14 (25.5)	24 (43.6)	17 (30.9)	38 (69.1)
5 years after the surgery	47	11 (23.4)	20 (42.6)	16 (34.0)	31 (66.0)

Complete autonomic control: complete control during the day and at night without the need for a urinal pad. Satisfactory control: the need for only one pad during the day or at night. Unsatisfactory control: requiring two or more pads during the day or at night

**Table 5:** Urodynamics of patients following spiral orthotopic ileal neobladder

Urodynamics	1 year after the surgery (*n* = 66)	3 years after the surgery (*n* = 35)	5 years after the surgery (*n* = 27)
Maximum bladder capacity (mL)	373.5 ± 46.3	385.6 ± 50.1	406.6 ± 55.3*
Pressure at maximum capacity (cmH_2_O)	26.6 ± 6.2	23.2 ± 4.3*	20.0 ± 3.9*^#^
Compliance (mL/cmH_2_O)	41.2 ± 6.9	40.5 ± 5.1	38.8 ± 4.5*
Maximum flow rate (mL/s)	12.5 ± 1.9	12.9 ± 1.7	14.9 ± 4.2*^#^
Pressure at maximum flow rate (cmH_2_O)	28.6 ± 9.3	28.2 ± 10.2	27.0 ± 8.6
Postvoid residual urine (mL)	14.0 (5.0, 89.0)	17.0 (11.0, 95.2)*	18.0 (13.7, 106.0)*^#^

Compared with 1 year after the surgery, *P < 0.05. Compared with 3 years after the surgery, ^#^*P* < 0.05

### HR-QoL

As shown in Table 6, the total FACT-BL scores of patients at 1, 3, and 5 years postoperation were 125.0 ± 15.2, 127.0 ± 16.2, and 120.6 ± 13.5, respectively. There was no significant difference in total FACT-BL scores between 1 year and 3 years postoperation, while the score decreased at 5 years postoperation (*P* < 0.05).

**Table 6:** Quality-of-life of patients following spiral orthotopic ileal neobladder

FACT-BL score	1 year after the surgery (*n* = 70)	3 years after the surgery (*n* = 55)	5 years after the surgery (*n* = 47)
PWB (0–28)	24.5 ± 2.1	25.1 ± 1.6	24.8 ± 1.7
SWB (0–28)	23.3 ± 3.0	23.0 ± 3.2	21.6 ± 3.3
EWB (0–24)	21.6 ± 2.7	20.4 ± 3.0	18.2 ± 2.4
FWB (0–28)	24.1 ± 2.3	24.0 ± 3.5	23.5 ± 2.6
BSS (0–48)	33.5 ± 5.2	34.5 ± 4.7	32.5 ± 4.7
Total (0–156)	127.0 ± 15.2	127.0 ± 16.2	120.6 ± 13.5*

*PWB* physical well being, *SWB* social/family well being, *EWB* emotional well being, *FWB* functional well being, *BSS* bladder cancer-specific scale

Compared with 1 year after the surgery, *P < 0.05

## Discussion

Orthotopic ureteral reimplantation has developed rapidly in recent years, and clinicians are gradually promoting the use of orthotopic neobladder surgery in clinical practice. In several medical centers, the proportion of orthotopic neobladder surgery after bladder resection has increased to 50–90%. Ileal neobladder is one of the most common methods of orthotopic neobladder surgery. At our center, approximately 50% of patients chose ileal neobladder diversion after radical cystectomy. This surgical technique provides acceptable functional outcomes for patients with the advantage of allowing transurethral access to the upper urinary tract. However, it also has some disadvantages that need to be overcome.

Controlling the complications of ileal neobladder surgery is a critical issue that needs to be addressed as it greatly affects the QoL of patients. Previous studies have shown that the complication rate of ileal neobladder surgery following radical cystectomy was as high as 28–58% [9–11]. A follow-up of 5 years was performed in the present study. The early complication rate was 47.2% and included infection (12.5%), diarrhea (6.9%), small intestine obstruction (4.2%), and catheter sepsis (4.2%); these complications were mainly caused by radical cystectomy surgery. The late complication rate was 58.3% and included ileourethral stenosis (18.1%), recurrent urinary tract infection (15.3%), and nocturnal incontinence (5.5%); these complications were mainly caused by urinary diversion surgery. These results were consistent with the report of International Consultation on Urological Diseases-International Society of Urology (ICUD-SIU) International Consultation [12]. Any form of urinary diversion surgery has its specific complications; in addition to surgical skills and experience of the surgeon, a regular long-term follow-up and prompt symptomatic intervention are necessary to avoid or reduce morbidity. In addition, the ileum is one of the satisfactory bladder replacements closest to the normal physiological state of the human body, but its secretory function is also an important cause of metabolic disorder syndromes [13]. After a sufficient curettage of the secretions, we used anhydrous ethanol to clean the intestinal mucosa to destroy its structure and reduce its absorption and secretion functions. Satisfactory results were obtained in the prevention of urinary mucus obstruction and absorptive metabolic disorder, and the incidence of urinary leakage was also reduced.

Previous studies indicated that the overall 5-year survival rate of urinary diversion following radical cystectomy ranged from 50 to 77.2% [14, 15], and the 10-year survival rate ranged from 38 to 66% [16, 17]. The overall survival rates of patients in our study were 97.2%, 76.4%, and 65.3% at 1, 3, and 5 years after surgery, respectively. All patients completed the urinary status survey, and some of them participated in the urodynamic studies. We found that incontinence was common in 3 months after surgery, but relieved over time. The total satisfactory control rate was 72.9%, 69.1%, and 66.0% at 1, 3, and 5 years after the surgery, respectively, and there was no statistically significant difference in the control rate at 1 year and 5 years postoperation. In terms of urodynamics, few parameters have been reported to be closely related to patient outcomes [18, 19]. For example, it is believed that severe damage would occur once post void residual urine exceeds 300 mL, which could also act as a trigger for urinary tract infection, calculi, and ureteral reflux [20]. In our study, the median of post void residual urine was 56 mL (range 47–106 mL), and no ureteral reflux was found in patients; however, we found that post void residual urine had a prognostic value for death at 3 and 5 years after surgery (ROC = 0.837 and 0.717, respectively). Considering that urination after urinary diversion is mainly driven by abdominal pressure, abdominal training is essential. We recommend that patients urinate every hour during daytime and every 2 h during nighttime with the help of an alarm clock. While urinating, the patients should relax their pelvic floor muscle, slightly increase abdominal pressure, bend forward, and perform palm presses to empty the bladder. Hautmann et al. [21] stated that high compliance levels are associated with the preservation of the upper urinary tract. Singh et al. [22] believed that high compliance is the main condition for achieving near-normal voiding patterns and preserving the upper urinary tract. In the present study, we found that compliance was another predictor for death at 3 and 5 years after surgery (ROC = 0.800 and 0.796, respectively).

Several questionnaires have been used to assess HR-QoL of patients with bladder cancer in clinics, and each questionnaire varies in its development, validation, and applicability to certain disease states [23]. FACT-BL consists of a functional assessment of cancer therapy—general scale (FACT-G V4.0) [24] and a bladder-specific scale of 12 questions; a higher score on FACT-BL reflects better HR-QoL. We found the total FACT-BL scores of patients at 1, 3, and 5 years postoperation were 125.0 ± 15.2, 127.0 ± 16.2, and 120.6 ± 13.5, respectively. There was no significant difference in the total FACT-BL score between 1 year and 3 years postoperation, while the score decreased at 5 years postoperation. Several reports have suggested that patients treated with ileal orthotopic neobladder after bladder resection have a better HR-QoL than those who underwent ileal conduit [25], while several other studies [26] reported contrary results. Cerruto et al. [27] reported comparable HR-QoL outcomes between the patients after ileal orthotopic neobladder and ileal conduit; this finding indicated that ileal orthotopic neobladder provided better results in some aspects of HR-QoL related to bowel function, but worsened urinary and sexual functions. It is difficult to confirm which type of diversion leads to a higher QoL, but most studies indicate that the HR-QoL of patients with bladder cancer decreased over time. The results of our present study support this conclusion, which indicates a regular long-term follow-up is necessary.

## Conclusions

In summary, we retrospectively analyzed the long-term outcome of 72 patients after an orthotopic ileal neobladder with orthotopic ureteral reimplantation. The complications of this procedure are relatively difficult to control, while its long-term functional and HR-QoL results are relatively satisfactory. We believe that a regular long-term follow-up is necessary to ensure appropriate HR-QoL of patients, and we also note that monitoring of urodynamics is beneficial for predicting long-term outcomes.
